# Hypertension from the patient’s perspective: contributions to the care offered by health professionals and self-care – a qualitative study

**DOI:** 10.1590/1516-3180.2022.0314.R1.17102022

**Published:** 2022-12-19

**Authors:** Felipe Leonardo, Clarissa Garcia Custódio, Débora Paulino de Lira, Dayana de Oliveira Ferreira, Maria Valéria Pavan, Fernando Antonio de Almeida

**Affiliations:** I Undergraduate Medicine Student, Faculty of Medical and Health Sciences, Pontifícia Universidade Católica de São Paulo (PUC-SP), Sorocaba (SP), Brazil.; II Undergraduate Medicine Student, Faculty of Medical and Health Sciences, Pontifícia Universidade Católica de São Paulo (PUC-SP), Sorocaba (SP), Brazil.; III Undergraduate Medicine Student, Faculty of Medical and Health Sciences, Pontifícia Universidade Católica de São Paulo (PUC-SP), Sorocaba (SP), Brazil.; IV Undergraduate Medicine Student, Faculty of Medical and Health Sciences, Pontifícia Universidade Católica de São Paulo (PUC-SP), Sorocaba (SP), Brazil.; V MD, PhD. Doctor-Assistant Professor, Department of Collective Health, Faculty of Medical and Health Sciences, Pontifícia Universidade Católica de São Paulo (PUC-SP), Sorocaba (SP), Brazil.; VI MD, PhD, Full Professor, Department of Clinics, Faculty of Medical and Health Sciences, Pontifícia Universidade Católica de São Paulo (PUC-SP), Sorocaba (SP), Brazil.

**Keywords:** Hypertension, Primary health care, Self care, Qualitative research, Health promotion, Disease prevention, Focal groups, Promotion of health

## Abstract

**BACKGROUND:**

Hypertension is the most common disease in primary care settings. Only 30% of cases were adequately controlled.

**OBJECTIVES:**

To analyze the knowledge and understanding of patients with hypertension regarding the factors that facilitate and limit adherence to treatment and, based on the results, build specific guidelines on hypertension self-care and control.

**DESIGN AND SETTING:**

This qualitative study was conducted in a primary healthcare setting.

**METHODS:**

Patients with hypertension who were followed at a primary healthcare unit were interviewed through focus groups, and a qualitative interpretation of their statements according to Bardin’s content analysis was performed.

**RESULTS:**

Three focus groups were formed (21 participants), from whose analysis emerged 74 core ideas related to the concept of hypertension, causes of increase in blood pressure, clinical consequences of hypertension, and possible patients’ contributions to help control blood pressure, arising from eating habits, psychosocial conditions, and lifestyle. Patients tend to accept the concept of “high blood pressure” as an inherent condition of the disease in their lives. Eating habits are strongly related to life history and self-perception of health. The association between high blood pressure and nervousness or stress appears to be strong.

**CONCLUSION:**

The experience of having “pressure problem” is unique for each person. It is necessary to optimize listening, recognizing that, for the patient to understand what hypertension is and its management, there must be understanding and convergence of proposals, adjustments, and changes in a positive and personalized way. As a result of this study, we implemented educational actions in primary healthcare units.

## INTRODUCTION

Hypertension, defined as the persistent elevation of systolic blood pressure (BP) to values ≥ 140 mmHg and/or diastolic BP ≥ 90 mmHg, is a chronic disease with a high worldwide prevalence.^1,2^ In Brazil, the disease affects 21.4–32.3% of the population, varying according to the survey’s methodological criteria.^2,3^ Among the risk factors for hypertension development are heredity, age, sex, race, overweight/obesity, excessive sodium consumption, sedentary lifestyle, excessive alcohol consumption, smoking, and socioeconomic factors, such as educational level.^2^ Hypertension is directly associated with a higher risk of cardiovascular and renal complications, particularly stroke, myocardial infarction, chronic kidney disease, and other serious complications.^2^ There is a slight increase in the mortality rate directly related to hypertension; however, 50% of deaths from cardiovascular disease are associated with hypertension.^2,4^

As the main objective of treatment is to reduce cardiovascular and renal morbidity and mortality associated with hypertension, the use of non-pharmacological and pharmacological treatment is essential in the reduction of BP levels and prevention of hypertension complications.^2^ In practice, non-pharmacological treatment mainly involves dietary measures aimed at weight loss and reduction of sodium consumption, regular physical activity, alcohol consumption reduction (when excessive), and smoking cessation. Regarding pharmacological treatment, adherence to medication is essential to achieve BP control.

Although the diagnosis of hypertension is relatively simple and some consider its treatment easy, the high percentage of individuals with undiagnosed hypertension and the number of patients diagnosed with hypertension who continue to have elevated BP express the complexity of monitoring these patients, requiring great attention and effort from the healthcare system, and there is no doubt about the importance of a multidisciplinary approach.^2^

Adherence to a therapeutic plan can be understood as the relationship between the guidance and prescription of the healthcare team and the patient’s conduct to follow them. Compliance with the therapeutic plan is a multidimensional process, which involves several circumstances: lack of knowledge about the disease and lack of motivation to comply with the treatment of a chronic disease; the absence of symptoms; unfavorable socioeconomic and educational background; difficulties in the relationship with healthcare services and in scheduling appointments; cost of certain medications and their adverse effects; and other aspects such as cultural and social beliefs and changes in quality of life after treatment initiation.^2,5^

Considering that a patient’s relationship with the healthcare team is an important element in the context of chronic diseases, a multidisciplinary approach in the follow-up of patients with hypertension, which focuses on the therapeutic process, is fundamental to the adherence and maintenance of treatment.^6^

In a primary healthcare unit (PHU) in a large city in São Paulo state, a pilot study is underway to evaluate a new systematization of care for the entire population with hypertension and/or diabetes mellitus accompanied by a family healthcare team (approximately 2,600 residents). This includes nursing care and home visits that aim for BP and glycemic control and individual and family guidance.

## OBJECTIVE

In the context of this pilot project, medical students working regularly at the PHU dedicated themselves to identifying the factors that facilitate and limit adherence to the treatment of hypertension in this population and understanding, from the patient’s perspective, the meaning and repercussions of the disease in their lives, contributing to the follow-up by the healthcare team.

## METHODS

This was an exploratory, qualitative, cross-sectional, analytical observational study. Individuals with hypertension belonging to the territory of a PHU with a family health strategy were invited to participate. The selection of participants with hypertension was aleatory among those attending the PHU. All the participants who agreed to participate were included in the study.

The areas of competence related to hypertension self-care were defined through a bibliographic survey in the Virtual Health Library database, considering articles written in Portuguese, English, and Spanish, from 2013 to 2018, using the descriptors “hypertension,” “self-care,” and “health education,” and involved the following topics: general notions about hypertension and its complications, BP self-control, self-care in drug treatment, self-care in the prevention of chronic and acute hypertension complications, and nutritional self-care. From this list of competencies, a script was formulated, which contained questions that guided the focus groups in analyzing patients with hypertension.

Focus groups were formed based on the definition proposed by Morgan: “A research technique that collects data through group interactions when discussing a special topic suggested by the researcher,” to understand feelings, beliefs, and participants’ behaviors.^7^ The focus group discussions were conducted until saturation of alternative answers was achieved and not pre-established. Three focus groups consisted of an average of seven participants per group. The discussions were recorded, transcribed, and analyzed by two authors, aiming to understand the most significant contents, according to the analysis process recommended by Bardin.^8^ The units were categorized according to the semantic criteria, establishing thematic categories that were exhaustively reviewed and described below.

The research project and informed consent form were submitted to a research ethics committee, approved on May 8, 2018, and only started after their approval (CAAE 86670318.6.0000.5373).

## RESULTS

A total of 21 participants with hypertension were included in the focus group. The participants’ characteristics are presented in Table 1. The sample of participants and their distribution by age, sex, autodeclared race or skin color, time from diagnosis of hypertension, BP levels, and number of antihypertensive prescriptions were similar to the profiles of those attending the PHU.

**Table 1.** Demographic and clinic characteristics of the participants

Parameter	Mean ± SD	Comments
Age	65.4 ± 13.6 years	> 60 years (70%)
Sex	13 women, 8 men	
Autodeclared race (skin color)	16 whites, 5 blacks	
Time from hypertension diagnosis	14.6 ± 8.7 years	
Systolic blood pressure	154.6 ± 18.3 mmHg	< 140/90 mmHg (33.3%)
Diastolic blood pressure	87.5 ± 11.6 mmHg	
Number of AH prescribed	Median = 2 AH	3 persons 1 AH12 persons 2 AH5 persons 3 AH1 person 4 AH

SD = standard deviation; AH = antihypertensive.

The content analysis allowed the classification of the speeches into categories in accordance with the core ideas, which summarized the topic addressed in the response. To analyze these core ideas, summary tables were prepared from the statements listed for each issue raised in the discussions. The different statements of the participants were considered and counted. Table 2 shows the core ideas based on the frequency of their appearance among the statements.

**Table 2.** Core ideas expressed during focus group discussion

Regarding the concept of hypertension or high blood pressure	Frequency
1. Cardiovascular diseases	5
2. Asymptomatic	4
3. Heredity	4
4. Unknown	3
5. Lifestyle	3
6. Related to the nervous system	2
7. Blood pressure levels	2
**Regarding possible causes of increased blood pressure**	**Frequency**
1. Nervousness/stress	17
2. Inadequate food intake	16
3. Salt consumption	10
4. Anxiety	5
5. Hypercholesterolemia/fat consumption	5
6. Consumption of alcoholic beverages and smoking	4
7. White coat hypertension	3
8. Treatment interruption	3
9. Sedentary lifestyle	3
10. Kidney injury	2
11. Weight excess	1
12. Others	4
**Regarding consequences of high blood pressure**	**Frequency**
1. Circulatory problems	5
2. Stroke/paralysis/dementia	4
3. Nephropathy	4
4. Neck pain	3
5. Myocardial infarction	3
6. Dizziness	3
7. Death	2
**Regarding control or prevention of high blood pressure**	**Frequency**
1. Healthy eating/salt reduction/water intake	54
2. Medicine treatment	47
3. Physical exercise	16
4. Access to health/information services	14
5. Emotional control	13
6. Self-care	5
7. Faith	4

Regarding the first question, “Does anyone know what hypertension or ‘high blood pressure’ is?” some representative expressions of the participants’ understanding of what hypertension is were as follows: “It is a silent disease, I don’t feel anything,” which is related to the absence of symptoms; “It must be a blood problem, right doctor? Change the heartbeat”; and “It messes with the circulation,” which is related to the core idea of cardiovascular disease.

Regarding the second question, ‘What can cause hypertension or high blood pressure?’ some examples of how they expressed themselves during the focus groups were as follows: “It’s an unwelcome inheritance, it’s hereditary,” which is related to the heredity of hypertension; “It’s what you eat, what you drink, and what you do during your life, right?,” which is related to dietary habits and lifestyle; “Pressure is nervous, stuff like that”; “The increase in blood pressure … has a lot of emotional influence, a lot of,” which is related to the core idea of nervousness, stress, and/or the nervous system.

Regarding the third question, “What are the consequences of high blood pressure?” the consequences were mentioned in a similar way to those illustrated in the following examples, which were obtained from their statements: “It can really cause kidney problems”; “Oh, it causes stroke, [hypertension] can cause a heart attack”; and “My mother died suddenly of a heart attack at age 47, from high blood pressure.”

In view of the nature of the answers, analyses of the fourth and fifth questions were grouped. The fourth question was “What can you do to control high blood pressure?” and the fifth was “What can you do to prevent high blood pressure?” Some statement on what helps hypertension control were as follows: “to consume little salt, and eliminate nervousness helps a lot”; “that’s where I think physical activity comes in, right?”; “oh… exercise, walking, medication…”; “you have to control the salt, which is the main thing, … and control the nerves”; “The only thing I do, my whole life, I’ve been taking medication for about 18 years, I take it all together, in the morning and at night.”

## DISCUSSION

Focus group discussion, the method selected for this study, provides the appreciation of the individual within the collective, under the influence of social pressure and the reactions that demand it, being in an intermediate position between pure observation and in-depth interviews.^9^

In brief, the results showed that the participants expressed the concept that hypertension is an asymptomatic disease caused by hereditary and behavioral factors, particularly dietary habits and lifestyle, and is associated with circulatory and renal diseases and complications. This was also evident in their statements regarding the understanding of emotional factors as possible causes of high BP. Regarding the control of hypertension, they valued adherence to adequate eating habits, physical exercise, emotional control, drug treatment, and access to health services. Self-care and faith were rarely mentioned but were present in their statements. Overweight or obesity was mentioned as a possible cause of hypertension only once. Difficulties in accessing the healthcare system or lack of antihypertensive medications did not emerge as problems in hypertension care.

Although the definition, awareness of causes, and recognition of complications of hypertension have been mentioned, these concepts were confused and mixed in their statements. Some participants provided the definition of “high blood pressure” as a pressure value achieved by assessing it with measuring devices. Others related it to a problem with the heart or blood vessels, while others associated it with lifestyle habits. Some studies have already shown that most patients do not know how to define or wrongly define hypertension.^10-12^ Renovato and Dantas claim that there is a “crisis of understanding” about the disease by patients with hypertension.^13^ These authors suggest that the absence of symptoms at the time when the healthcare professional imputes him as sick is responsible for the contradiction, while the constant high BP levels throughout the disease follow-up end up convincing them that there is really something wrong, making them move along this gradient of conviction about being carriers of this “hypertension” entity.^13^

In addition to this difficulty in recognizing the problem, there is still semantic disagreement in the discourse between doctors and patients. Fleischer, in her ethnographic study conducted in Guariroba (Federal District) on patients with hypertension, recognized the disparity in meanings between the terms used by doctors and what the patient understands.^14^ The nosological category “hypertension” does not exactly match “high blood pressure” in popular vocabulary but the term “pressure problem.”^14^ This is due to the fact that patients link the moments of uncontrolled BP, which are represented by pressure peaks, to the term “high blood pressure” and understand this phenomenon as transitory, often related to the emotional status, as arising from a moment of stress. Conversely, having the disease “under control,” without documented moments of pressure peaks, is better understood by patients as a “pressure problem.” This is preceded by the verb “to have,” presenting a permanent character, while “high blood pressure” is usually preceded by the verb “to be/*estar*,” evidencing its transience.^14^

Our study also showed that, as a group, patients tended to assume the definition of “high blood pressure” as the practical condition that the disease has in the life of each of them and not on a biomedical basis as was expected by the researchers, which allows us to understand that, in the participant’s view, the full concept of hypertension did not matter only in terms of meaning for them but in what it would pragmatically reflect, that is, its consequences (stroke, heart attack, kidney problems, circulatory problems, and even death). Moreover, symptoms such as neck pain, dizziness, leg pain, shortness of breath, and tachycardia have been reported, which, from a physician’s perspective, will only be present after the complications of hypertension arise.^1,2^ It is evident in the speech of the study participants and in the literature that there is a misconception that these reported symptoms represent hypertension itself and not common complications of the advanced stage of the disease, which are preventable with adequate BP control.^10-12^

Participants also mentioned the need for chronic food restriction and drug treatment for the rest of their lives, factors recognized in the literature for influencing quality of life and adherence to treatment.^15,16^ Food intake is strongly linked to the history of people’s lives, culture, and self-perception of health. Many people have a history of vulnerability or food deprivation at other times in their lives and are negatively associated with restrictions required by their disease at such times, which makes their adherence difficult. The absence of visible signs of the disease in their daily lives makes them question the real need to adopt such habits as well as influence adherence to drug treatment.^17^

In the quest to identify the origin of their health problems, participants recognized factors such as high consumption of salt, fatty foods, alcoholic beverages, and cigarettes; sedentary lifestyle; and heredity as present in the genesis of hypertension. Such factors have already been reported by patients in other studies, and the result was not surprising.^10^ However, it is noteworthy that there was only one mention of overweight and obesity as risk factors for hypertension. Although there were several references to inadequate nutrition as a possible cause of the disease, the participants did not relate carbohydrate excess and weight gain as a risk factor for hypertension nor did they recognize the limitation of the consumption of carbohydrates and fat as a way of reducing weight and consequently BP. We were also surprised by the overestimation of the etiological association between hypertension and nervousness, which were mentioned in terms such as “nervous system,” “nervous,” “nerve,” “emotional,” “stress,” “anxiety,” and “frightening.” Such a relationship has already been demonstrated in other studies, although it is unclear whether the association made by patients is a cause or a consequence.^10,11,13,17-20^ It is assumed that patients perceive the disease as a thermometer of their emotional state, oscillating along with their emotions, even treating the two things as the same entity.^16^ The two things are so strongly aggregated in the participants’ imagination that they assume as valid the strategy that it is possible to control the disease just by controlling nervousness, even evoking a curative characteristic of hypertension if the emotions that trigger it were controlled.^14,19,21^

Fava et al. and Fleischer discussed in their ethnographic studies how this hypertension–nervousness marriage is taken into account by healthcare professionals who take care of these patients.^14,22^ Although there are physiological bases on the contribution of stress in the development of high BP, the hypertension control programs of our healthcare system do not include effective strategies to deal with these factors.^14,22^ In fact, in many places of the Unified Health System, it is difficult to adequately discuss and treat mental health disorders, a topic shrouded in taboo, which allows the perpetuation of myths and prejudices when referring to the topic of hypertension.^14^

In addition to controlling the emotional state, patients understand that the modification of lifestyle recognized as causes of high BP is a way of exercising self-care and controlling BP. However, this is a challenge in practice. The chronic nature of the treatment is one of the reasons that leads to the discontinuation of these practices, causing discouragement, since the practices that need correction are often the same ones that produce a psychologically compensatory effect for the individuals, considered by them as protectors of the stress that they believe to be the cause of the increase in their BP.^15,17^ Moreover, the state of social vulnerability that most patients are in makes it difficult to access healthier life practices.^20^

Health professionals have an opportunity to intervene in these groups of people, aiming to spread techniques that are economically viable and easily accessible, so that these people can find qualified scientific information.

Adherence to pharmacological treatment is ambiguous, as it is frequently associated with the occurrence of symptoms, which are often late in this group. In the absence of BP peaks and symptoms, patients with hypertension carry a conception of having been cured, allowing themselves to interrupt the treatment, even with the opposite orientation.^16^

As a result of this study and with the support of medical students who attend the PHU, we are implementing several actions in health units, such as forming support networks via social media that can offer health education, exchanging information, and facilitating the follow-up of patients with hypertension; distributing educational posters and leaflets in PHU with short and direct information; encouraging self-care and self-measurement of BP at home; and establishing simple and direct nutritional guidelines. Some examples of sentences with information and guidelines were communicated:

“Did you know that the best medicine to fight the pressure problem is to lose weight?”

“People with pressure problem don’t feel any discomfort, but it can hurt their heart, brain, and kidneys.”

“If you have pressure problem, learn how to measure your blood pressure at home with automatic (arm) devices. It is very simple and cheap and helps to control your blood pressure.”

“If you have high blood pressure and you take medication to control it, keep taking them, even if you have had alcoholic beverages.”

“You can help control your blood pressure. Eats with little salt, lose weight if they are overweight, and perform regular physical exercise (walking is the best).”

Due to the coronavirus disease 2019 pandemic, we should still avoid crowds to prevent disease transmission; however, as soon as sanitary conditions allow, group meetings will be promoted to encourage self-care educational practices for patients with hypertension.

## CONCLUSION

The group approach of patients with hypertension allowed us to analyze how the experience of having “pressure problem” is unique for each person. Being personal, it is not possible to imagine an awareness strategy that can reach everyone. It is important to confirm in this study the perception that these patients need professionals who listen to them, seeking to recognize the meanings of their speeches, in contrast to the use of scientific and biomedical jargon, through pre-shaped recipes for a human being impersonal and generalized. For this, it is necessary to listen to these patients, with a high degree of empathy, recognizing that, for the patient to understand what hypertension is and its management, there must be understanding, convergence of proposals, adjustments, and changes in a positive and personalized way.
